# Triboelectric nanogenerators for neural data interpretation: bridging multi-sensing interfaces with neuromorphic and deep learning paradigms

**DOI:** 10.3389/fncom.2025.1691017

**Published:** 2025-11-07

**Authors:** Lingli Gan, Shuqin Yuan, Min Guo, Qian Wang, Zongfang Deng, Bin Jia

**Affiliations:** Center for Neurology, The Thirteenth People’s Hospital of Chongqing, Chongqing, China

**Keywords:** triboelectric nanogenerators, neural data interpretation, deep learning, spiking neural networks, neuromorphic computing, brain–computer interfaces, elderly health monitoring

## Abstract

The rapid growth of computational neuroscience and brain–computer interface (BCI) technologies require efficient, scalable, and biologically compatible approaches for neural data acquisition and interpretation. Traditional sensors and signal processing pipelines often struggle with the high dimensionality, temporal variability, and noise inherent in neural signals, particularly in elderly populations where continuous monitoring is essential. Triboelectric nanogenerators (TENGs), as self-powered and flexible multi-sensing devices, offer a promising avenue for capturing neural-related biophysical signals such as electroencephalography (EEG), electromyography (EMG), and cardiorespiratory dynamics. Their low-power and wearable characteristics make them suitable for long-term health and neurocognitive monitoring. When combined with deep learning models—including convolutional neural networks (CNNs), recurrent neural networks (RNNs), and spiking neural networks (SNNs)—TENG-generated signals can be efficiently decoded, enabling insights into neural states, cognitive functions, and disease progression. Furthermore, neuromorphic computing paradigms provide an energy-efficient and biologically inspired framework that naturally aligns with the event-driven characteristics of TENG outputs. This mini review highlights the convergence of TENG-based sensing, deep learning algorithms, and neuromorphic systems for neural data interpretation. We discuss recent progress, challenges, and future perspectives, with an emphasis on applications in computational neuroscience, neurorehabilitation, and elderly health care.

## 1 The imperative for advanced healthcare monitoring in an aging world

The interpretation of neural data represents one of the most critical challenges in modern computational neuroscience and a cornerstone for the future of personalized medicine. Brain signals are inherently high-dimensional, nonlinear, and noisy, with complex temporal dependencies that complicate analysis (Farias et al., 2018). Traditional statistical and signal processing approaches often fail to capture the intricate dynamics of neural activity, limiting their effectiveness for long-term monitoring and clinical applications, particularly in real-world, uncontrolled environments (Meng et al., 2023).

This analytical challenge is compounded by a pressing societal need. The world is experiencing an unprecedented demographic shift, with a rapidly aging population (Lutz and Kc, 2010; Mahmood and Dhakal, 2023). This trend brings a rising prevalence of age-related conditions, including neurodegenerative and cognitive disorders (Ogugua et al., 2024), placing a significant strain on healthcare systems and caregivers (Lampersberger et al., 2023; Stimpfel et al., 2020). The growing demand for continuous, long-term neural monitoring requires new sensing and computational frameworks that can move healthcare from the clinic to the home (Fick, 2021; World Health Organization, 2020). To be effective, especially for elderly care, these technologies must be wearable, low-power, and biologically compatible (Anghel et al., 2020; He and Lee, 2021), extending beyond conventional cardiovascular and metabolic parameters to include nuanced neural and cognitive functions (Nasr et al., 2021). However, the reliance on batteries for power remains a significant hurdle, limiting the practicality, reliability, and sustainability of long-term wearable devices (Chan et al., 2009; Wu et al., 2020).

In this context, triboelectric nanogenerators (TENGs), originally developed for energy harvesting, have emerged as transformative candidates for multi-sensing in biomedical and neural applications (Zhu et al., 2020; Dong et al., 2020). By efficiently converting ambient mechanical energy from sources like human motion into electricity, TENGs offer a path to self-powered operation (Lai et al., 2022; Pandey et al., 2024), thereby eliminating the dependence on external power sources. Their inherent advantages—including mechanical flexibility, material versatility (Anwer et al., 2022; Yuan et al., 2023), and the ability to perform multimodal signal acquisition (Zhuo and Sun, 2020; Méndez et al., 2020)—make them exceptionally suitable for capturing a diverse array of biophysical signals. This includes crucial neural activity-related measures such as electroencephalography (EEG) and electromyography (EMG), as well as other vital signs like pulse wave (Yao et al., 2023; Chen et al., 2021), respiration (Liu et al., 2019), and pressure (Bai et al., 2014), creating a holistic view of a patient’s health status. However, the rich, multi-stream data generated by these TENG-based sensor systems requires advanced computational paradigms for effective interpretation (Meng et al., 2023; Wu et al., 2021).

**Figure 1:**
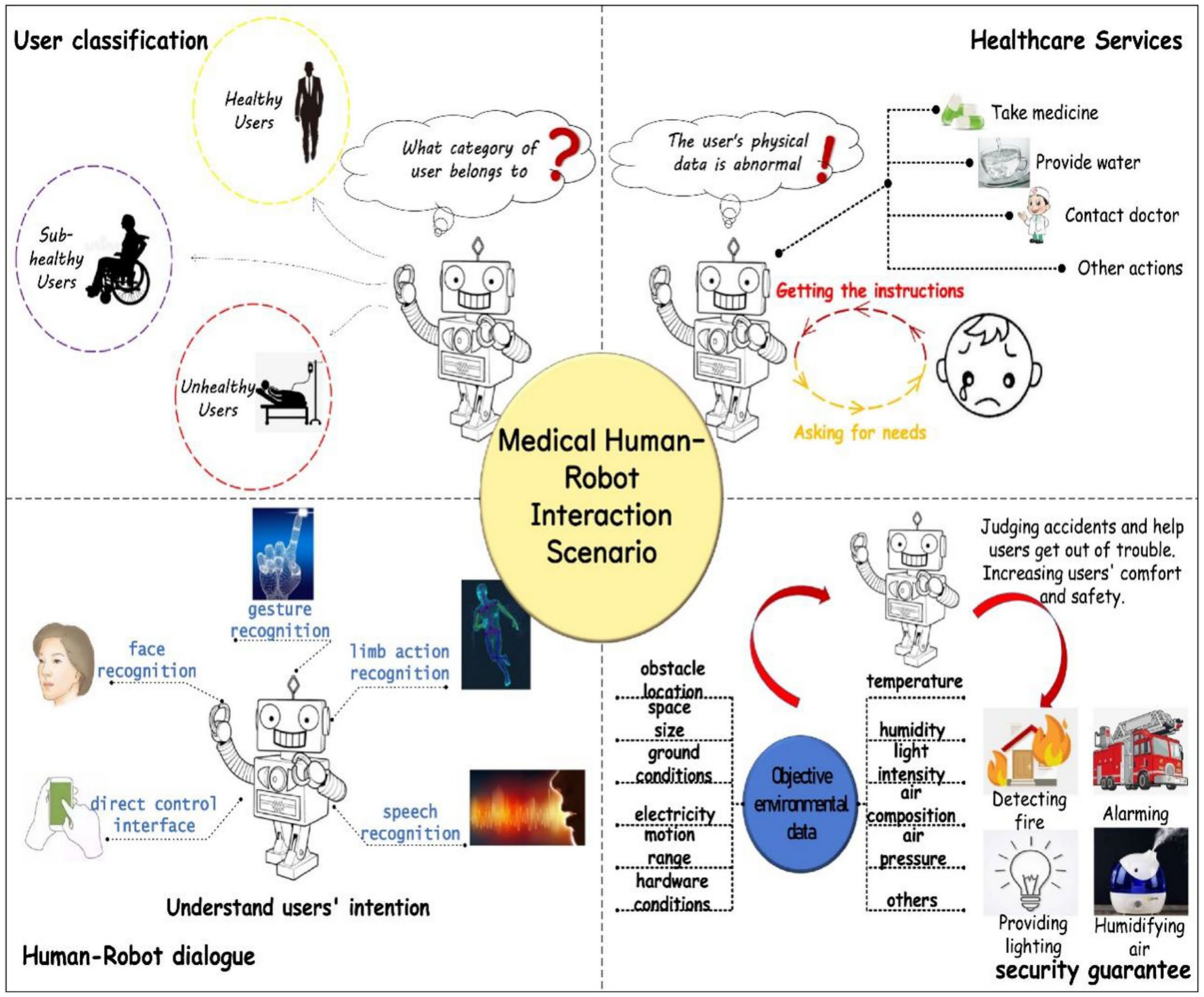
Medical Human-Machine Interaction Scenario Service (Lin et al., 2020).

Recent advances in deep learning and neuromorphic computing provide powerful new opportunities to decode TENG-acquired neural and physiological signals (Nasr et al., 2021; Lin et al., 2020). Deep learning models, such as Convolutional Neural Networks (CNNs) and Recurrent Neural Networks (RNNs), have shown remarkable promise in classifying brain states, detecting cognitive impairments (Farias et al., 2018), and interpreting complex neural connectivity patterns. These AI-driven approaches are essential for transforming raw sensor data into actionable clinical insights (Méndez et al., 2020; Nasr et al., 2021). As illustrated in Figure 1, the integration of such AI with multi-sensor networks creates a powerful human-machine interaction framework for elderly health (Lin et al., 2020; Anghel et al., 2020). This system can provide personalized health recommendations through real-time monitoring, thereby alleviating caregiver burdens (Lampersberger et al., 2023; Franck et al., 2016) and significantly improving the quality of life for seniors (Ogugua et al., 2024; Fick, 2021).

The integration of Triboelectric Nanogenerator-based multi-sensor systems and artificial intelligence (AI) in elderly health monitoring can significantly enhance accuracy and real-time capabilities (Meng et al., 2023; Wu et al., 2021) while alleviating caregiver burdens (Stimpfel et al., 2020) and improving the quality of life for seniors (Franck et al., 2016; Anghel et al., 2020). Consequently, thorough research on self-powered multi-sensor and AI technologies in this context is both practically significant and holds substantial development potential (Nasr et al., 2021; Luo et al., 2023). Multi-sensor network technology can track physical performance indicators (Kulurkar et al., 2023; Mahdi et al., 2021), and when paired with deep learning for data processing (Meng et al., 2023; Méndez et al., 2020), it offers innovative solutions for elderly health monitoring. Triboelectric nanogenerators efficiently convert mechanical energy from the environment into electrical energy (Zhu et al., 2020; Lai et al., 2022), providing a sustainable power source for sensors, which enhances system convenience and sustainability (Wu et al., 2020; Bulathsinghala et al., 2023). The multi-sensor system gathers real-time physiological, environmental, and behavioral data from the elderly through various sensors (Chan et al., 2009; Zhuo and Sun, 2020), creating a comprehensive health monitoring network. AI technologies, particularly machine learning and deep learning, can analyze this extensive data to identify health risks, provide early warnings (Farias et al., 2018; Nasr et al., 2021), and deliver personalized health recommendations (Méndez et al., 2020; Lin et al., 2020).

**Figure 2:**
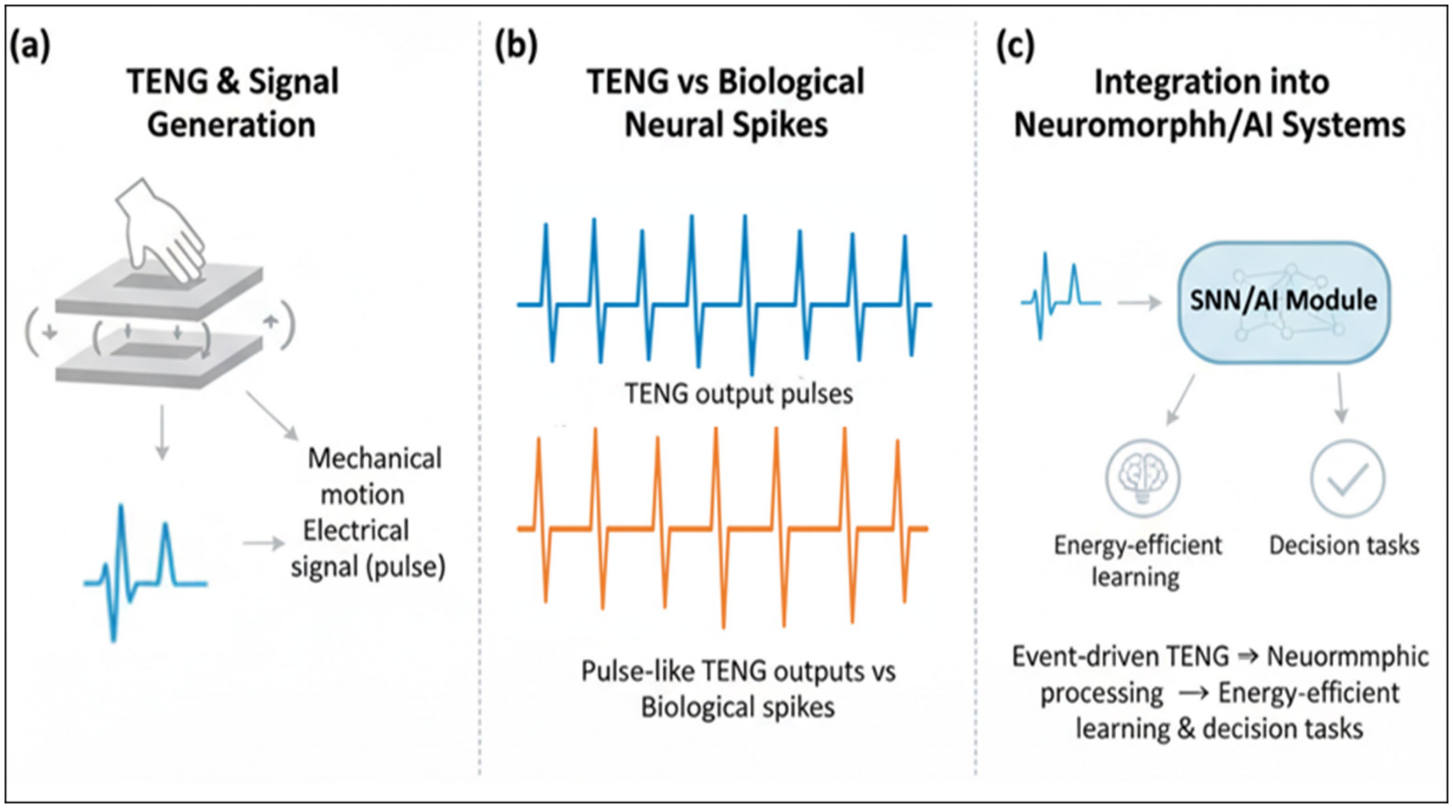
Conceptual framework linking TENG sensing and computational paradigms. **(a)** Schematic of TENG and the signal-transformation pipeline from mechanical motion to raw electrical outputs. **(b)** Comparison between TENG pulse-like outputs and biological neural spikes. **(c)** Integration of event-driven TENG signals into neuromorphic processing pipelines (SNN/AI) for energy-efficient learning and decision tasks.

Beyond traditional deep learning, the field is moving toward more biologically inspired models. Spiking neural networks (SNNs) and neuromorphic hardware architectures offer an event-driven, energy-efficient computational paradigm (Nasr et al., 2021) that naturally aligns with the sparse and discrete nature of both biological neural signals and TENG sensor outputs (Lin et al., 2020). This synergy promises a new generation of ultra-low-power biomedical devices capable of sophisticated on-device data processing (He and Lee, 2021; Koo et al., 2020) (Figure 2 illustrates how TENG-generated pulse-like signals can be seamlessly mapped into neuromorphic frameworks for event-driven computation).

In this mini-review, we examine the pivotal role of TENG-based sensing in computational neuroscience (Wu et al., 2020; Zhang et al., 2021) and discuss how its convergence with deep learning and neuromorphic paradigms can profoundly enhance neural data interpretation (Nasr et al., 2021; Lin et al., 2020). We highlight current progress in developing these integrated systems (Meng et al., 2023; Wu et al., 2021), outline key challenges related to signal fidelity, system integration, and data analysis (Brunner, 2023; Lu et al., 2023), and present a forward-looking perspective for the future integration of TENGs into advanced neural monitoring and brain–computer interface applications (He and Lee, 2021; Wang et al., 2022). To make this perspective accessible across disciplines, Figure 2 provides a conceptual overview linking TENG signal generation, preprocessing, and downstream AI-driven interpretation pathways. Beyond elderly healthcare, TENG–AI systems have also demonstrated potential in diverse domains such as intelligent prosthetics, athletic performance monitoring, environmental sensing, and soft robotic perception.

## 2 TENG-based multi-sensing interfaces for neural data acquisition

Triboelectric nanogenerators (TENGs) have emerged as a transformative technology for neural monitoring, leveraging the fundamental principles of contact electrification and electrostatic induction to create highly versatile sensing platforms (Zhu et al., 2020; Dong et al., 2020). Their unique capability to directly convert biomechanical energy into measurable electrical signals enables truly self-powered operation, addressing one of the most significant limitations in long-term neural and physiological monitoring systems (Lai et al., 2022; Bulathsinghala et al., 2023). Unlike conventional sensors that remain constrained by the finite lifetime of battery power sources (Chan et al., 2009; Wu et al., 2020), TENG-based systems offer inherent advantages for both wearable and implantable applications (Anghel et al., 2020; He and Lee, 2021), positioning them as a key enabling technology for next-generation computational neuroscience and neurorehabilitation solutions (Nasr et al., 2021; Lin et al., 2020).

The material versatility and structural design flexibility of TENGs allow for acquisition of a broad spectrum of biophysical signals with direct relevance to neural activity (Anwer et al., 2022; Zhang et al., 2021). For electroencephalography (EEG) and electromyography (EMG) applications, while traditional approaches rely on measuring biopotentials directly, TENG-based systems can capture the subtle mechanical motions of the skin and underlying tissues that accompany these electrical activities (Pang et al., 2020; Han et al., 2023). These self-powered sensors can be fabricated from soft, biocompatible polymers to create conformal interfaces that improve user comfort and signal stability compared to conventional rigid electrodes (Yuan et al., 2023; Rahman et al., 2024). The mechanical energy harvested from scalp movements or muscle contractions generates electrical signals that faithfully reflect underlying neuromuscular activity, enabling new approaches to motor control studies and brain-muscle interaction analysis (Lin et al., 2020; Luo et al., 2023).

In cardiorespiratory monitoring, TENGs demonstrate exceptional sensitivity to the pressure variations and vibrations associated with cardiovascular and pulmonary function (Bai et al., 2014; Liu et al., 2019). The autonomic nervous system’s tight regulation of these physiological processes makes them valuable proxies for cognitive states and stress responses. Textile-integrated TENG sensors can monitor pulse waves with high fidelity (Chen et al., 2021), while chest-mounted patches track respiratory patterns through thoracic expansion measurements (Bai et al., 2014). These capabilities provide crucial neural context without requiring complex, power-intensive equipment (Casey et al., 2020; World Health Organization, 2020), representing a significant advancement in ambulatory monitoring technologies.

The application of TENGs extends to tactile and motion sensing, where their high sensitivity to pressure and strain enables detailed tracking of limb movement, gait dynamics, and postural control (Kulurkar et al., 2023; Mahdi et al., 2021). As illustrated in Figure 2, tactile sensor arrays based on TENG technology can effectively mimic the functional properties of human skin, with particular relevance for prosthetic limb applications (Ha et al., 2018; Chang et al., 2023). These systems provide not only monitoring capabilities but also closed-loop sensory feedback, offering new possibilities for understanding the neural control of movement and developing advanced neurorehabilitation strategies (Méndez et al., 2020; Lin et al., 2020). Collectively, these examples underscore how TENGs function as multimodal neural interfaces, simultaneously capturing diverse physiological signals in a self-powered manner. Figure 2 provides a representative overview of these multimodal sensing capabilities and their relevance for computational neuroscience applications.

**Figure 3:**
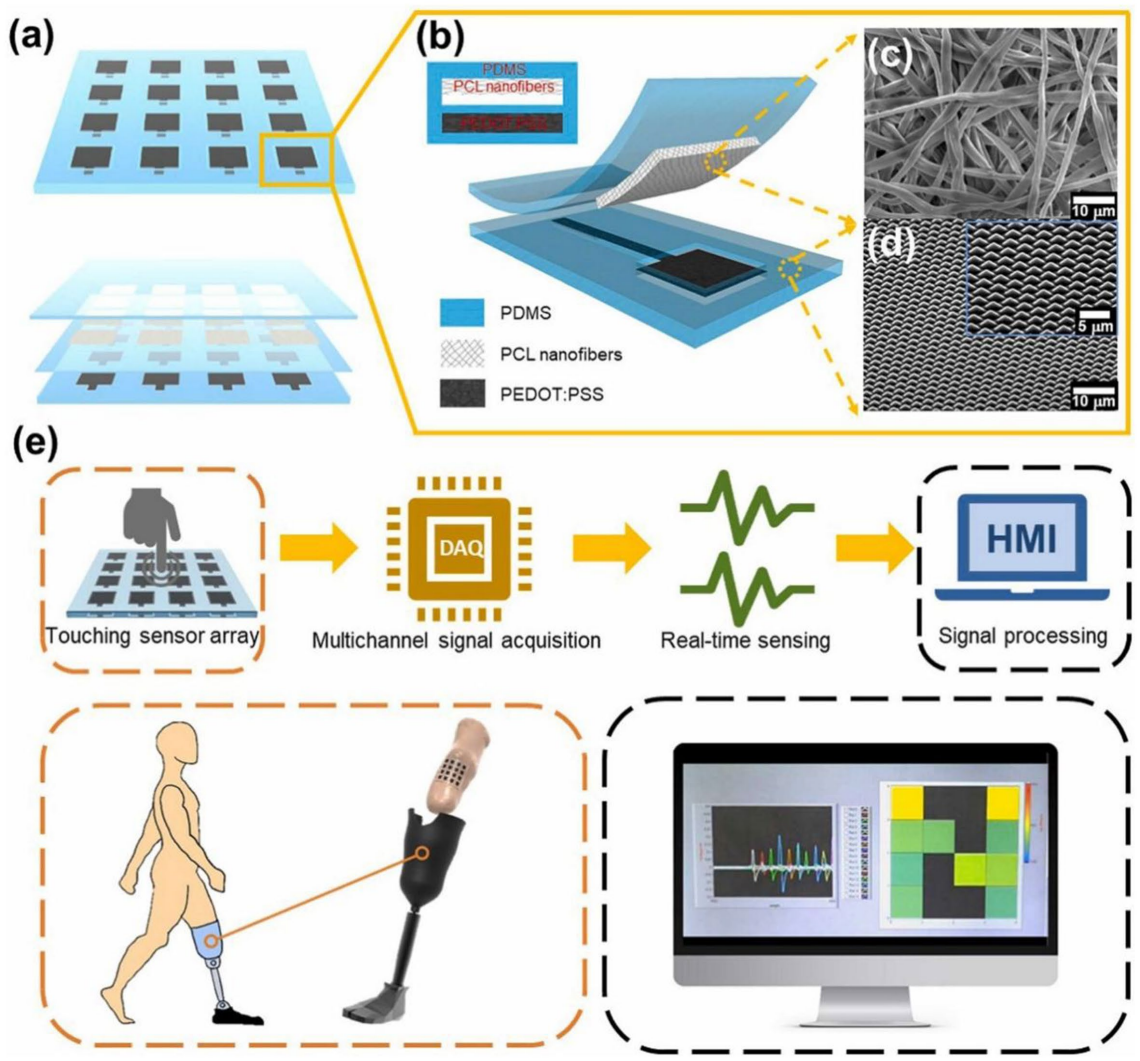
**(a)** Schematic diagram of the tactile sensor array system. **(b)** Cross-sectional layered view of the tactile sensor based on PDMS, PCL nanofiber membrane, and PEDOT electrodes. **(c)** SEM image of the PCL nanofiber membrane. **(d)** SEM image of the pyramid-patterned PDMS layer. **(e)** Schematic diagram of the integration of the tactile sensor array system with a multi-channel data acquisition system and a relative pixel representation of the pressure distribution inside the prosthetic socket displayed on the HMI system.

Several defining characteristics establish TENGs as a disruptive force in neural interface technologies. The self-powered nature of these devices eliminates dependence on external energy sources, enabling sustainable long-term operation through continuous harvesting of biomechanical energy (Lai et al., 2022; Pandey et al., 2024; Bulathsinghala et al., 2023). Material flexibility allows for the creation of soft, conformal interfaces using biocompatible polymers and hydrogels (Anwer et al., 2022; Rahman et al., 2024), significantly improving wearability and reducing motion artifacts compared to conventional rigid sensors (He and Lee, 2021; Han et al., 2023). A single TENG device can be engineered for multimodal operation, simultaneously detecting diverse physiological parameters such as pressure, strain, and vibration (Zhuo and Sun, 2020; Wu et al., 2021), thereby providing a more comprehensive view of complex brain–body interactions than traditional single-modality sensors (Nasr et al., 2021; Lin et al., 2020). The inherently scalable fabrication processes support both miniaturization for implantable applications and development of large-area, high-density sensor arrays (Dong et al., 2020; Ha et al., 2018), as demonstrated in Figure 3, making the technology adaptable to various monitoring scenarios.

From a computational neuroscience perspective, TENGs represent more than just a novel sensing modality—they provide an interface that fundamentally connects physical neural activity with advanced computational frameworks (Nasr et al., 2021; Lin et al., 2020). The high-dimensional spatiotemporal data generated by multi-channel TENG arrays offers rich input streams for deep learning architectures, enabling sophisticated analysis of brain states and motor intent decoding (Farias et al., 2018; Méndez et al., 2020; Luo et al., 2023). Notably, the characteristic pulse-like signals produced by many TENG designs bear a striking resemblance to the spiking activity of biological neurons (Nasr et al., 2021). This inherent compatibility with neuromorphic processing paradigms, including spiking neural networks (SNNs) and event-based processors (Lin et al., 2020), enables highly efficient neural data interpretation that mirrors the brain’s own remarkable computational efficiency (He and Lee, 2021; Koo et al., 2020). This synergy between TENG-based sensing and biologically inspired computing architectures points toward a future where neural monitoring systems can achieve unprecedented levels of performance and energy efficiency (Zhang et al., 2021; Wang et al., 2022).

Recent experimental studies have verified the feasibility of TENG-based electrodes for acquiring physiological and neural signals, including ECG, EEG, and EMG (Pu et al., 2016; Huang et al., 2025; Yang et al., 2023). Comparative evaluations with conventional Ag/AgCl electrodes indicate that TENG-based sensors can reproduce the characteristic waveforms of these signals with acceptable fidelity. For instance, EEG recordings captured by TENG electrodes preserve the dominant *δ*, *θ*, *α*, and *β* rhythms, although slight reductions in signal-to-noise ratio (SNR) and high-frequency fidelity have been observed compared to Ag/AgCl electrodes (Huang et al., 2025). Similarly, TENG-based ECG and EMG monitoring demonstrates clear waveform morphology aligned with traditional electrodes, validating their capability for neural and neuromuscular signal acquisition (Pu et al., 2016; Yang et al., 2023).

Rather than aiming to replace conventional electrodes in terms of precision, the primary advantages of TENG-based neural interfaces lie in their self-powered operation, mechanical flexibility, and conformability to the skin. These features minimize motion artifacts, enhance user comfort, and enable continuous, long-term monitoring in wearable or implantable systems. Therefore, TENGs should be regarded as complementary to existing technologies, offering unique benefits for sustained and multimodal neural data acquisition in computational neuroscience applications.

## 3 Deep learning for TENG-based neural data interpretation

The advent of deep learning has revolutionized analysis of complex biological signals, uncovering spatiotemporal patterns that elude conventional signal processing methods (Meng et al., 2023; Nasr et al., 2021). This computational paradigm becomes particularly powerful when applied to the multimodal data streams generated by triboelectric nanogenerator (TENG) systems, enabling translation of raw sensor data into clinically actionable insights for vulnerable populations including the elderly and neurologically impaired patients (Méndez et al., 2020; Lin et al., 2020). The marriage of TENG-based sensing with deep learning forms the technological foundation for next-generation smart healthcare systems capable of real-time neural and physiological monitoring (Wu et al., 2021; Nasr et al., 2021).

Convolutional Neural Networks (CNNs) have demonstrated remarkable efficacy in processing TENG-acquired EEG and EMG signals due to their innate capacity for spatial feature extraction (Nasr et al., 2021; Lin et al., 2020). By transforming time-series data into 2D representations such as spectrograms or connectivity matrices, CNNs can identify discriminative patterns for diverse classification tasks. These tasks include detecting neurological states (sleep stages, attention levels) and recognizing pathological signatures such as epileptic discharges (Farias et al., 2018; Meng et al., 2023).

For geriatric applications, CNN-based models enable differentiation of cognitive impairment profiles using TENG-derived signals. This serves both clinical diagnostics and fundamental neuroscience research, highlighting the translational potential of integrating TENG signals with AI-driven analysis (Farias et al., 2018; Nasr et al., 2021).”

The inherently sequential nature of neural data demands architectures capable of modeling temporal dependencies - a role fulfilled by Recurrent Neural Networks (RNNs) and their Long Short-Term Memory (LSTM) variants (Nasr et al., 2021; Lin et al., 2020). These networks excel at decoding the dynamic transitions in TENG signals that reflect brain–body interactions, providing critical insights into phenomena like cognitive fatigue progression or stress response dynamics (Méndez et al., 2020; Luo et al., 2023). Hybrid CNN-RNN architectures combine these strengths, employing CNNs for spatial feature extraction at each timestep while RNNs analyze temporal evolution (Lin et al., 2020). This dual approach enhances performance in complex applications including motor intention prediction and brain-computer interface control (Méndez et al., 2020; Lin et al., 2020), making deep learning indispensable for interpreting continuous TENG data streams.

TENG technology’s unique capability for simultaneous multimodal signal acquisition (EEG, EMG, cardiovascular, motion) finds its ideal computational counterpart in deep learning-based multimodal fusion (Zhuo and Sun, 2020; Wu et al., 2021). By learning the complex interrelationships between disparate physiological systems, these models achieve superior diagnostic accuracy compared to single-modality analysis (Nasr et al., 2021; Lin et al., 2020). For elderly care, such integrated analysis enables earlier detection of neurodegenerative conditions and more accurate fall risk assessment through combined evaluation of cardiorespiratory, movement, and neural signals (Kulurkar et al., 2023; Mahdi et al., 2021). This multimodal approach moves beyond isolated measurements to construct a comprehensive picture of an individual’s health status (Meng et al., 2023; Méndez et al., 2020).

Despite these advances, significant challenges persist in deploying deep learning for TENG data interpretation. The scarcity of high-quality, well-annotated TENG neural datasets limits model generalizability across diverse populations (Nasr et al., 2021; Morley et al., 2020). The opaque “black-box” nature of many deep learning systems raises interpretability concerns that may hinder clinical adoption (Nasr et al., 2021; Morley et al., 2020). Most critically, the substantial computational demands of traditional deep learning architectures conflict with the low-power design philosophy essential for wearable TENG systems (Lin et al., 2020; He and Lee, 2021). These challenges are driving exploration of brain-inspired neuromorphic approaches that promise to combine analytical power with energy efficiency and interpretability (Nasr et al., 2021; Lin et al., 2020; Koo et al., 2020), potentially overcoming current limitations to enable widespread deployment of intelligent TENG-based monitoring systems.

**Figure 4:**
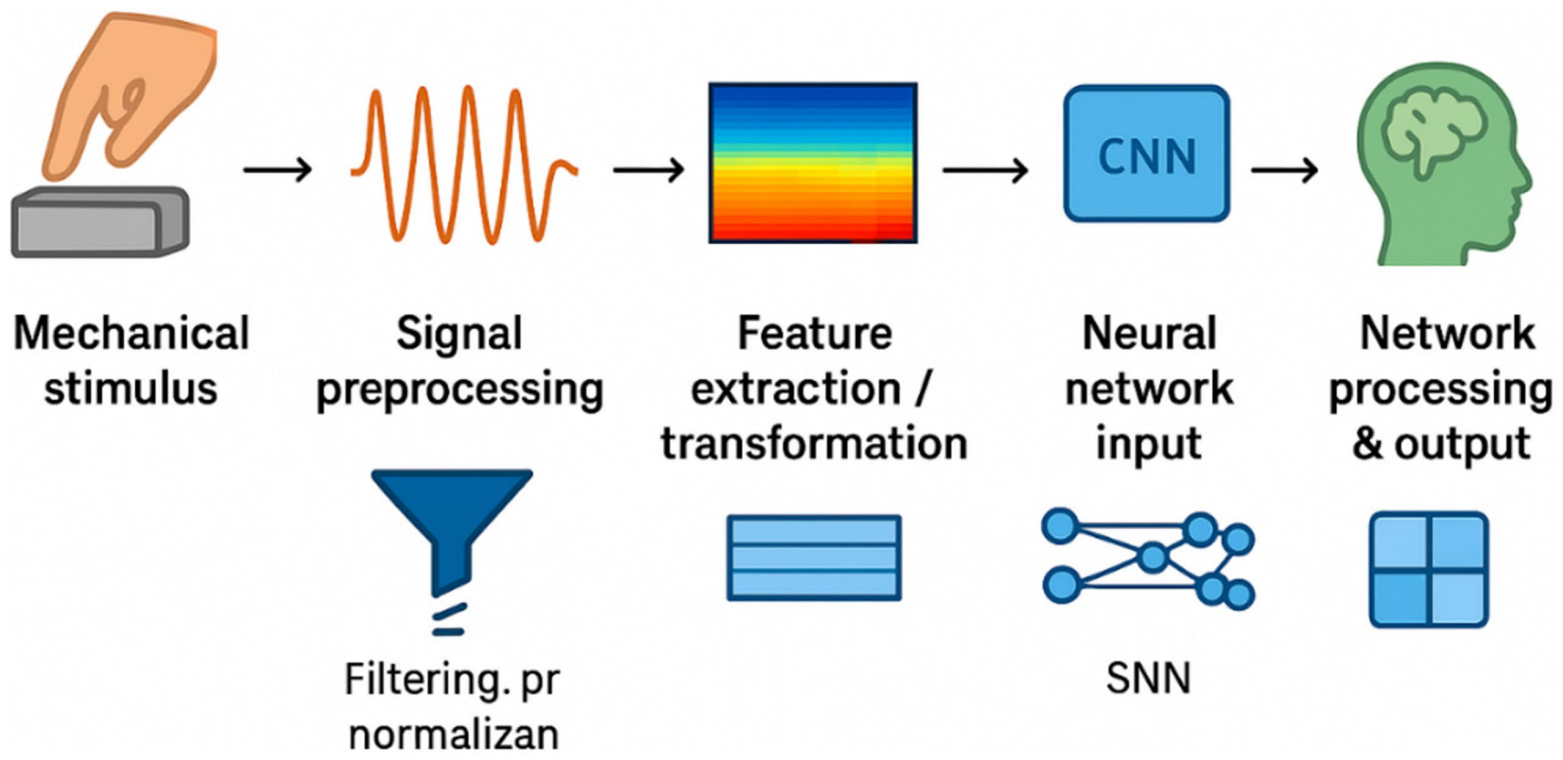
Workflow of TENG signal acquisition and neural network processing.

To illustrate the practical workflow, Figure 4 summarizes a typical processing pipeline for TENG-acquired signals before entering deep learning models. Raw pulse-like signals generated by TENGs are first filtered and normalized to reduce noise and baseline drift. The preprocessed data can then be transformed into time–frequency representations (e.g., spectrograms) or structured time series features. These representations serve as inputs for neural networks, where convolutional neural networks (CNNs) are effective for extracting spatial–frequency patterns, and recurrent neural networks (RNNs) capture temporal dependencies. Such pipelines have already been applied in preliminary studies of motion detection (Fan et al., 2012), electrocardiogram recognition (Pu et al., 2017), and activity quantification (Zhang, 2024) using TENG-based sensors, demonstrating the feasibility of integrating TENG signals with machine learning frameworks for physiological and neural data interpretation.

## 4 Neuromorphic paradigms for energy-efficient neural sensing

While deep learning has revolutionized neural data analysis, its computational demands create fundamental limitations for real-time wearable applications (Nasr et al., 2021; Lin et al., 2020). The energy-intensive nature of conventional artificial neural networks directly contradicts the design objectives of unobtrusive, long-term monitoring systems (He and Lee, 2021; Koo et al., 2020). Neuromorphic computing emerges as a transformative alternative, offering biologically plausible processing that aligns seamlessly with both the operational principles of biological neurons and the signal characteristics of triboelectric nanogenerators (TENGs) (Nasr et al., 2021; Lin et al., 2020). This paradigm shift from continuous computation to event-driven processing enables autonomous healthcare systems that combine intelligent analysis with unprecedented energy efficiency (Lin et al., 2020; Koo et al., 2020).

Spiking Neural Networks (SNNs) represent the computational foundation of this approach, mimicking the temporal dynamics of biological neurons through discrete, asynchronous spike events (Nasr et al., 2021). Their event-driven operation provides three key advantages for TENG integration: (1) drastic reduction in computational overhead by processing only when input changes occur, (2) native compatibility with the pulsed output characteristics of many TENG designs, and (3) inherent capacity to model temporal patterns in neural oscillations and autonomic nervous system activity (Lin et al., 2020; Koo et al., 2020). This synergy eliminates power-hungry signal conversion steps, as TENG-generated pulses can directly modulate SNN activity (Lin et al., 2020; Lai et al., 2022). When implemented on neuromorphic hardware, SNNs achieve real-time performance for applications ranging from motor intent decoding to closed-loop neurostimulation (Nasr et al., 2021; He and Lee, 2021).

The hardware embodiment of this paradigm has seen remarkable progress through platforms like Intel’s Loihi and IBM’s TrueNorth (Lin et al., 2020; Koo et al., 2020). These neuromorphic processors execute spike-based computations with orders-of-magnitude greater energy efficiency than conventional architectures (He and Lee, 2021). Their parallel, distributed design enables compact wearable systems where TENGs provide both sensory input and supplemental power (Lai et al., 2022; Bulathsinghala et al., 2023), while neuromorphic chips perform edge-based signal processing (Lin et al., 2020). This co-design approach yields multiple system-level benefits: (1) elimination of cloud dependency reduces latency to milliseconds, (2) on-device processing enhances data privacy for sensitive health information, and (3) distributed architecture efficiently handles multimodal data from TENG arrays (Zhuo and Sun, 2020; Lin et al., 2020; Koo et al., 2020).

The TENG-neuromorphic convergence creates unique opportunities for autonomous neural interfaces (He and Lee, 2021; Wang et al., 2022). Energy harvested through body movement powers both sensing and processing, forming self-sustaining feedback loops ideal for real-time applications (Lai et al., 2022; Bulathsinghala et al., 2023). In elderly care scenarios, such systems could continuously monitor cognitive state and fall risk without user intervention (Kulurkar et al., 2023; Mahdi et al., 2021), while neurorehabilitation applications benefit from instantaneous biofeedback during therapy sessions (Méndez et al., 2020; Lin et al., 2020). The pulsed operation of both TENGs and SNNs enables temporal coding schemes that further optimize energy use (Nasr et al., 2021; Lai et al., 2022).

Current limitations facing this integration include the relative immaturity of SNN training algorithms compared to deep learning methods (Nasr et al., 2021; Lin et al., 2020), limited accessibility of commercial neuromorphic hardware (Koo et al., 2020), and the need for closer sensor-processor co-design (He and Lee, 2021). However, rapid advances in materials science (Anwer et al., 2022; Zhang et al., 2021) and neuromorphic engineering (Lin et al., 2020; Koo et al., 2020) are addressing these challenges. The inherent compatibility between TENG sensing and spike-based processing suggests this bioinspired approach will play a central role in future wearable neural interfaces (Nasr et al., 2021; He and Lee, 2021), potentially enabling continuous monitoring systems that operate for years without battery replacement (Lai et al., 2022; Bulathsinghala et al., 2023).

## 5 Challenges and future perspectives

The integration of triboelectric nanogenerators (TENGs) with deep learning and neuromorphic paradigms presents a transformative opportunity for neural data interpretation, promising a future of autonomous, personalized healthcare. However, before this vision can be fully realized, several key challenges spanning from fundamental material science to clinical ethics must be systematically addressed. These hurdles represent the critical frontiers where future research and innovation are most needed.

### 5.1 Signal quality and standardization

A foundational challenge lies in the signal quality and standardization of TENG-based sensors. While TENGs offer the unparalleled advantages of self-powered operation and multimodal sensing, their electrical outputs can be highly sensitive to environmental conditions such as humidity, material degradation over time, and the inherent variability of biomechanical motion. These factors can introduce significant noise and inconsistency into neural-related data acquisition, posing a major obstacle to achieving the reproducibility and reliability required for clinical applications. To ensure that data is comparable across studies and individuals, the field urgently needs standardized fabrication methods, robust calibration protocols, and advanced signal preprocessing pipelines designed to denoise and normalize TENG outputs.

### 5.2 Data availability and algorithmic development

Beyond the raw signal, the “fuel” for deep learning models—large, well-annotated datasets—remains exceptionally scarce. Effective training of robust AI models requires vast amounts of high-quality, labeled data, yet publicly available datasets collected with TENG-based neural sensors are extremely limited. The process of manually annotating continuous neural data is not only labor-intensive and time-consuming but also requires domain expertise and is often subjective. This data bottleneck is perhaps the single greatest barrier to progress. To overcome it, the community must foster collaborative data-sharing initiatives. Furthermore, exploring semi-supervised or self-supervised learning methods will be critical to leverage the large volumes of unlabeled TENG-derived data that can be collected more easily.

At a conceptual level, a central tension exists between the high performance of conventional deep learning and its significant drawbacks in biological plausibility and computational cost. While neuromorphic computing directly addresses these concerns with its brain-inspired efficiency, its algorithmic maturity currently lags behind that of deep learning. A critical area for future research is the development of hybrid models that strategically combine the powerful representational learning of deep networks with the efficiency and temporal processing strengths of neuromorphic approaches. Such models could achieve a crucial balance, advancing computational neuroscience by creating tools that are both powerful and interpretable.

### 5.3 Toward clinical translation

To truly unlock the potential of these integrated systems, a paradigm shift toward holistic hardware-sensor co-design is essential. Realizing the full synergy between TENGs and neuromorphic systems requires strategies where sensors, algorithms, and hardware are optimized in concert rather than in isolation. For instance, TENG device geometries could be tailored to generate outputs that are intrinsically spike-like, allowing them to communicate in the native language of SNNs and thereby reduce preprocessing overhead. Similarly, designing neuromorphic chips with input channels that can directly interface with the high-impedance, capacitive nature of TENGs could enable the creation of highly efficient, closed-loop neural monitoring and stimulation systems.

The success of TENG-based neural interfaces will ultimately depend on clinical translation and societal acceptance. Applications in elderly health monitoring and neurorehabilitation introduce challenges related to data privacy, long-term biocompatibility, and rigorous clinical validation.

Ensuring patient and clinician trust requires careful attention to safety and regulatory compliance. Ethical frameworks should be proactively developed to address autonomy, informed consent, and potential algorithmic bias, especially when deploying these systems in vulnerable populations.”

Looking forward, the roadmap for this field is rich with promising research directions. Future work should focus on multimodal integration, combining TENG-based signals with traditional electrophysiology, medical imaging, and behavioral data to build more holistic models of brain states. The development of hybrid neuromorphic-deep learning computational paradigms will be essential for balancing efficiency with accuracy. Pushing intelligence to the edge by embedding AI models directly into TENG-powered wearable systems will enable real-time, autonomous neural monitoring. Perhaps most excitingly, this could lead to the creation of neuroadaptive systems—intelligent feedback loops where TENG-AI systems not only monitor but also actively modulate neural activity for therapeutic purposes. By addressing these challenges head-on, the fusion of TENG-based sensing, deep learning, and neuromorphic computing is poised to pave the way for a new generation of intelligent, energy-efficient, and biologically inspired systems for neural data interpretation. In addition to healthcare-oriented applications, TENG–AI integration also faces broader challenges such as signal variability under dynamic motion, cross-domain generalization, and seamless integration with robotic or environmental monitoring platforms.

## 6 Conclusion

The convergence of triboelectric nanogenerators (TENGs), deep learning, and neuromorphic computing represents a promising and synergistic frontier in computational neuroscience and personalized healthcare. This mini-review has highlighted how TENGs provide a self-powered, flexible, and multimodal sensing platform capable of capturing a wide range of physiological and neural-related signals, offering significant advantages in comfort, sustainability, and versatility over conventional electrodes and sensors. When these rich data streams are coupled with advanced deep learning methods, the complex, noisy signals can be effectively decoded into meaningful insights about brain states, neural connectivity, and cognitive health. This is particularly transformative for applications such as elderly care, where continuous, unobtrusive monitoring is crucial for the early detection of cognitive decline and other age-related conditions.

At the same time, the inherent limitations of deep learning—namely its high computational and energy costs—are directly addressed by the emerging field of neuromorphic computing. This brain-inspired paradigm offers an energy-efficient and biologically plausible alternative for real-time neural data interpretation. The characteristically spike-like, event-driven nature of TENG outputs aligns naturally with the operational principles of Spiking Neural Networks and neuromorphic hardware, creating a seamless pathway toward scalable, ultra-low-power neural monitoring systems that can perform complex analysis at the edge. Together, these technologies provide complementary strengths: deep learning contributes unparalleled pattern recognition capabilities for offline and high-accuracy analysis, while neuromorphic computing delivers the on-device efficiency and biological plausibility essential for continuous, real-time applications.

Looking ahead, the thoughtful integration of TENG-based sensing with these AI-driven computational frameworks is poised to reshape how neural data are acquired, processed, and interpreted. Such integrated systems hold the potential to profoundly advance brain-computer interfaces, accelerate progress in neurorehabilitation technologies, and revolutionize remote and elderly health monitoring. More broadly, this interdisciplinary approach embodies a new paradigm for decoding brain function, one that closes the loop between material innovation, artificial intelligence, and computational neuroscience. By bridging this gap, we can move closer to a future of intelligent, autonomous, and deeply personalized neurological healthcare.
